# Disordered dolomite as an unusual biomineralization product found in the center of a natural *Cassis* pearl

**DOI:** 10.1371/journal.pone.0284295

**Published:** 2023-04-26

**Authors:** Chunhui Zhou, Shiyun Jin, Ziyin Sun, Artitaya Homkrajae, Elina Myagkaya, Nanthaporn Nilpetploy, Kwanreun Lawanwong

**Affiliations:** 1 Research Department, Gemological Institute of America (GIA), New York, New York, United States of America; 2 Research Department, Gemological Institute of America (GIA), Carlsbad, California, United States of America; 3 Pearl Identification Department, Gemological Institute of America (GIA), Carlsbad, California, United States of America; 4 Pearl Identification Department, Gemological Institute of America (GIA), Bangkok, Thailand; Saveetha Institute of Medical and Technical Sciences: Saveetha University, INDIA

## Abstract

Natural pearls are produced without human intervention, mainly due to various irritations from the surrounding environment to their mantle tissues. Pearls usually possess similar mineral compositions to the host shells, which means they are also dominated by aragonite and calcite. In this study, we report a natural pearl from a *Cassis* species mollusk containing granular central structures. Raman spectroscopy, laser ablation inductively coupled plasma mass spectrometry (LA-ICPMS), energy dispersive X-ray spectroscopy (EDS) coupled with scanning electron microscope (SEM), and X-ray diffraction (XRD) analyses were carried out in order to characterize the mineral composition in the center region of this pearl. Our results showed that this pearl’s center was made of mostly disordered dolomite (Ca_0.53_Mg_0.47_CO_3_) mixing with small amount of aragonite and high magnesium-calcite. To the best of our knowledge, this is the first time disordered dolomite was conclusively identified inside of a natural pearl and such information expanded our knowledge on internal growth structures and formation of natural pearls.

## Introduction

Biomineralization of mollusk shells is a complex combination of biochemical and physiological processes. In addition to the growth of shells, some mollusk species in Bivalvia and Gastropoda classes can also produce pearls, a unique type of gem material that has been treasured by human beings since antiquity [1]. A natural pearl may form when an irritant get into the soft body of an oyster, mussel, clam, or sea snail, which causes their mantle tissues’ epithelial cells to secrete calcium carbonate to coat the irritant and eventually turns it into a pearl. Similar to their host shells, pearls are predominantly made of aragonite and calcite: two most thermodynamically stable forms of calcium carbonate. Other varieties of calcium carbonate structures such as vaterite, amorphous calcium carbonate, and high magnesium-calcite have also been found in either shells or pearls, although with much less frequency [2–8].

The formation and mineralization of pearls and shells have been previously studied by many researchers but the exact mechanism is still not completely understood. Most of the work involved in investigation of the nacre structures. According to Addadi and coworkers [9], the process of mollusk shell nacre formation can be described in the following order: organic matrix assembly, formation of the primary minerals (likely amorphous calcium carbonate), nucleation of nacre tablets, and the growth of the tablets. This process was believed to be heavily regulated by the organic matrix of the shell, which is composed of proteins, polysaccharides, lipids, pigments, free amino acids and peptides [10]. Similar finding on the formation of pearls has also been reported previously [11].

Due to overharvesting of natural oyster beds around the world, pearl industry shifted from natural pearl fishing to cultured pearl farming in the beginning of the 20^th^ century and cultured pearl products have dominated the market since then [12]. Gem quality natural pearls are still highly sought after and this continues to incentivize small-scale pearling activities in different parts of marine as well as freshwater environments around the globe in modern-day world [13–15]. In addition, natural pearls are also recovered as side products from shellfishing activities, which the original main goals are to fish for oyster (or other types of mollusks) meat and shells but pearls are also found unexpectedly during the processes.

Dolomite [CaMg(CO_3_)_2_] is one of the most abundant carbonate minerals in the Earth, mostly present in the Paleozoic and Precambrian sedimentary dolostone successions that are hundreds of meters in thickness [16]. However, despite being supersaturated in Mg, the carbonates precipitating from modern sea water are predominately aragonite and calcite, with very rare occurrences of dolomite. Moreover, extensive laboratory experiments have demonstrated that dolomite is extremely difficult to synthesize under the Earth surface conditions [17], likely due to the hydration barrier of Mg cations in the solution [18]. This discrepancy is widely known as the “dolomite problem”, which has been puzzling geologists for over a century. Dolomite is a major host for oil and gas reservoirs as well as ore deposits, and plays an important role in carbon sequestration, which may be critical in solving the climate challenges we are facing today. Tremendous efforts have been made solving the “dolomite problem”, by combining field observations, laboratory experiments and even atomistic computer simulations, but a complete answer has not been found yet.

In this study, we examined a natural pearl reportedly found in a *Cassis* species mollusk recovered by a fisherman in the areas of Indonesian waters. Belonging to the Cassidae family, *Cassis* species are commonly known as helmet shells and inhabit tropical and temperate oceans from intertidal to subtidal depths. Many species are harvested as bi-products in the fishing industry, sometimes serve as a food source in some parts of the world, and their beautiful shells have also been collected by divers and snorkelers. Very little information have been reported on the biominerlaization of the shells or pearls produced in these species, so this study gave us a unique and rare opportunity to examine the internal growth structures and biomineralization process of such materials. To our surprise, we discovered disordered dolomite in the center of this pearl, which was an unusual mineralization product from mollusks.

## Materials and methods

The pearl sample used in this study was provided to us by an Indonesian pearl dealer who visited our laboratory in 2015. It was reportedly found from a *Cassis* species mollusk in the areas of Indonesian waters. *Cassis* is a genus of very large sea snails (marine gastropods) that are also called helmet shells. Pearls have been occasionally found in these gastropods [19, 20], as well as submitted to us for identification in the past. This particular sample weighs 0.68 carat, exhibits a semi-baroque shape, yellowish brown coloration, and a typical non-nacreous surface appearance commonly seen in pearls produced from such species.

The sample was sawn in half using a SMART CUT ^™^ cutting machine from UKAM Industrial Superhard Tools company with a Lapcraft Dia-Laser^™^ diamond saw blade to reveal its internal structures. The smaller half was subsequently ground by a Sashi diamond lap 600# and polished by a Lightside ^™^ lap. Photomicrography of the interior features of the two cut samples was performed using a Nikon SMZ18 stereomicroscope with SHR Plan Apo 1× objective lens and NIS-Elements imaging software. Backscattered electrons (BSE) images were collected using a Zeiss EVO MA 10 scanning electron microscope (SEM), at 15 kV accelerating voltage and 1 nA probe current with a working distance of 8.5 mm.

A Renishaw inVia Reflex micro-Raman spectrometer system with a 50× magnification Leica objective lens and an 830 nm diode laser excitation wavelength at room temperature was used to analyze structural characteristics on various locations of the sample in a scanning range from 100 to 1600 cm^–1^. It is a common technique used to distinguish CaCO_3_ polymorphs (mainly between aragonite and calcite) with reference to different band positions of carbonate ion (CO_3_^2−^) modes. Raman mapping was also used to determine the distribution of the material made up the brown granular center and the white region around it on one of the cut samples. The mapping was performed using a Thermo Scientific DXRxi Raman imaging microscope (Madison, WI, USA) with 785 nm laser excitation wavelengths at room temperature, 0.055 sec exposure time per pixel, 25 μm pixel size, and a total of 500 scans. The very low exposure time was used due to the high background fluorescence of the sample. Thousands of spectra were collected across the area and visualized using a false color map of specific peak position.

A small fragment (~20 μm) was separated from the center of the pearl and mounted on a micro loop for XRD data collection. The data were collected on a 3 circle single-crystal X-ray diffractometer, which is equipped with a Rigaku 007 Cu-Kα X-ray source and a Bruker PROTEUM-F135 CCD detector. Three φ+ω-rotation frames were collected for 600s/frame with a detector distance of 150 mm and 2θ = 30°, 50°, 70°. The data were reduced to 1D powder diffraction pattern using APEX3 software. The crystal structure is refined using the Rietveld method against the integrated data in the Jana2006 program [21].

In-situ XRD data were collected directly on the pearl for a more comprehensive analysis of the different zones in the sample. A single-crystal X-ray diffractometer equipped with a Bruker MICROSTAR microfocus Cu-Kα rotating anode generator and an APEXII CCD detector, which produce much stronger X-ray beam with smaller divergence, was used to collect the data. The half pearl was glued to a capillary tube on the spherical natural surface, with the flat cut surface facing outward, perpendicular to the tube. The area of interest was centered at χ = 90°, while the ω was set to equal to θ (half of the 2θ detector position) to make the incident angle about the same as diffracted angle. Five φ-rotation frames were collected for 240s/frame for each spot, with a detector distance of 200 mm and 2θ = 15°, 30°, 45°, 60°, and 75° respectively. The frames were unwarped and merged using APEX3 software, and then integrated to Intensity-2θ powder diffraction patterns.

Chemical analyses were performed using a Thermo Fisher Scientific’s iCAP Qc ICP-MS, coupled with an Elemental Scientific Lasers NWR213 laser ablation system with a frequency quintupled Nd:YAG laser operated in Q-switched (pulsed) mode at a wavelength of 213nm and pulse duration of 4 ns. Detailed operation conditions for the laser and the ICP-MS instrument are the same as what was described in the previously published paper [22]. The chemical composition was initially internally standardized with 43Ca at a preset value of 400400 ppmw calculated and assumed from pure calcium carbonate. The data was then normalized and converted to 100 wt. % oxides based on 1 carbon and 3 oxygen atoms. In addition, energy dispersive X-ray spectroscopy (EDS) coupled with SEM was also employed to provide additional chemical analyses for the center region of the pearl. Sample was prepared for analysis by mounting on a metal stub and was then gold coated with a Cressington 108 Auto/SE Sputter Coater using a cycle of 15s to deposit a gold coating of roughly 10 nm. EDS mapping was collected using an Oxford X-MaxN 20 detector at a pixel dwell time of 60 microseconds and a resolution of 2048px. Both point and map analyses were collected at 15 kV accelerating voltage, 1 nA probe current, and a working distance of 8.5 mm.

The data that support the findings of this study are openly available in Zhou, Chunhui et al. (2022), Dataset for Disordered dolomite as an unusual biomineralization product found in the center of a natural Cassis pearl, Dryad, Dataset, https://doi.org/10.5061/dryad.v41ns1s13.

## Results and discussion

### General observations

**Fig 1:**
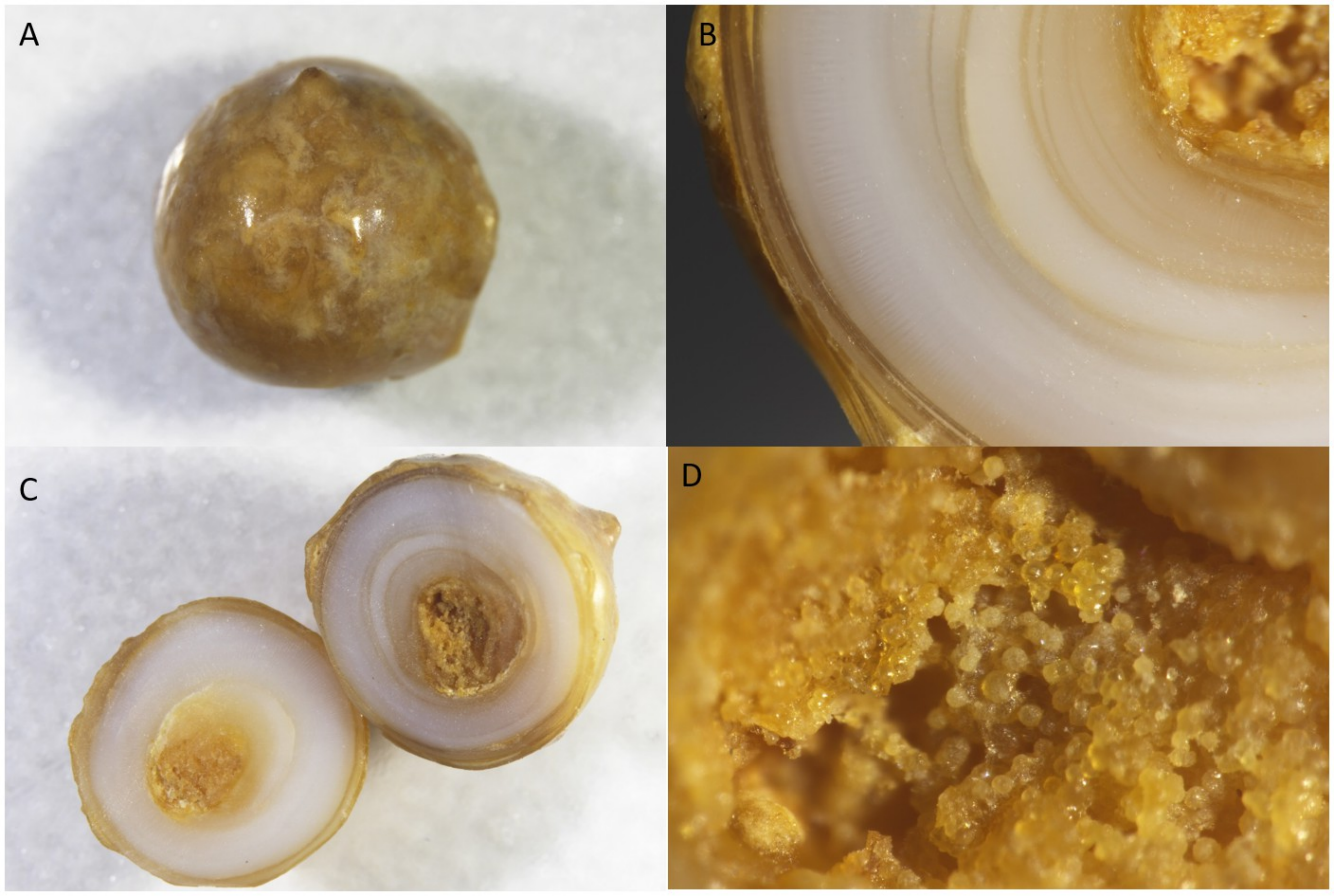
Macro- and microscopic images on the surface and cross-section of the pearl showing its various growth structures.

**Fig 2:**
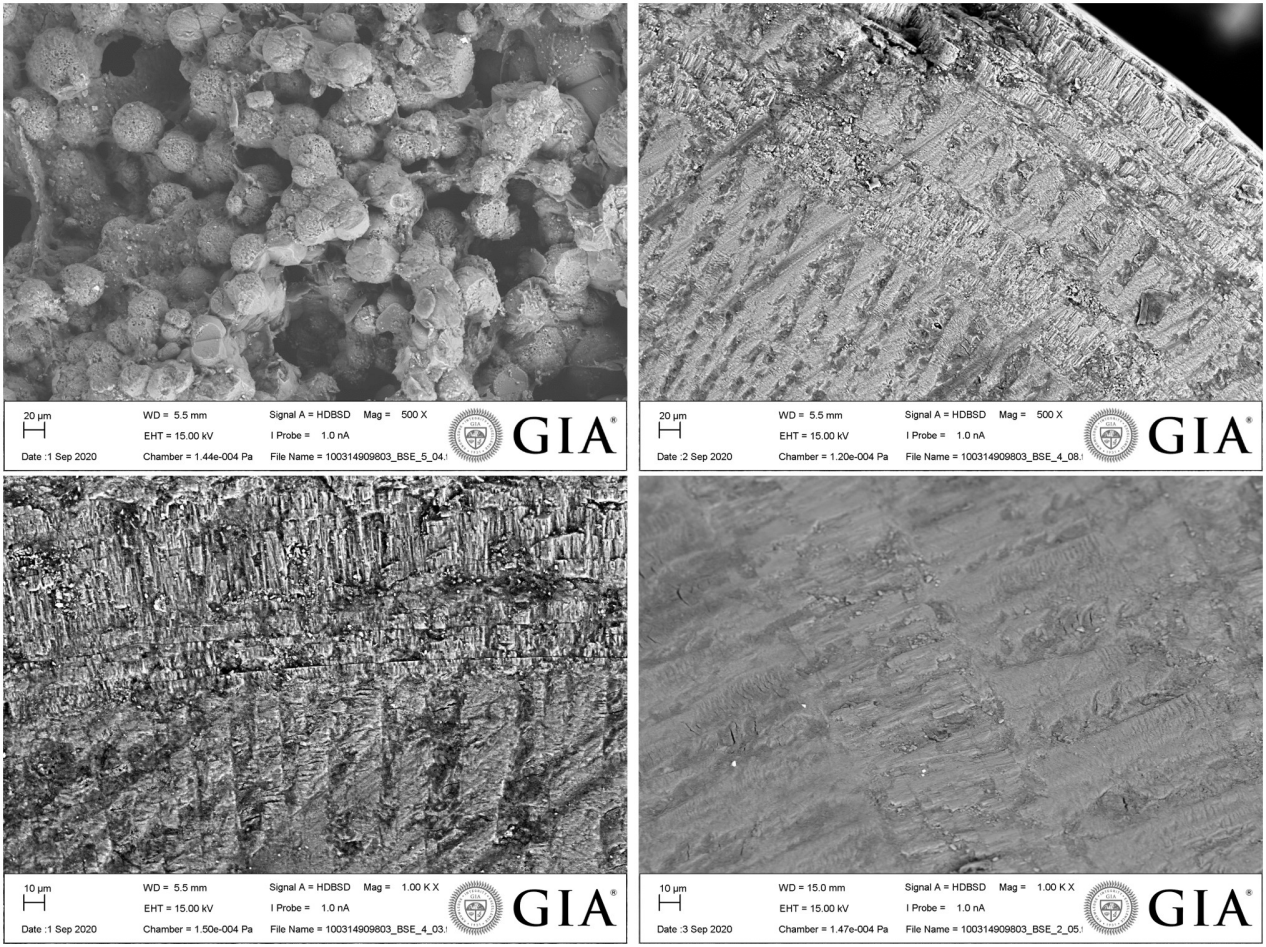
BSE images taken at the center (upper left), outer growth region (upper right), close-up image of the boundary area between outer white and brown growth regions (lower left), and close-up image of the brown outer growth region (lower right) of the pearl at its cross section.

Pearls can be either nacreous or non-nacreous, depending on how calcium carbonate crystallites are deposited throughout their structures. Nacreous pearls contain stacked aragonite tablets in parallel superimposition (often called the “brick-and-mortar” structure), while non-nacreous pearls often show a flame structure that is due to a crosswise and crisscross array of bundles of aragonite laths or fibers (also called the “plywood” structure) [23, 24]. Macro- and microscopic images on the surface and cross-section of the sample are shown in Fig 1. Its yellowish brown surface exhibited a mottled appearance lacking any aragonite platelet layers, suggesting it to be non-nacreous, which is expected for pearls formed in *Cassis* species. The cross-section showed multiple white growth zones with brownish growth rings, as well as subtle flame patterns due to their crossed-lamellar microstructures. More interestingly, the center region of this pearl had a distinct morphology and a porous appearance, revealing an area of numerous spheroidal aggregates with each having an approximate diameter ranging from 30 to 60 μm in size. Close-up images of the center and outer regions, including boundary area between white and brown outer regions at the sample’s cross-section taken by SEM are shown in Fig 2. From these images it is clear that its internal aragonite laths or fibers have alternative growth directions and orientations. It appears that this pearl has gone through several distinct growth stages with different biomineralization processes, and within each stage having certain mesoscale periodic structural patterns different from nacreous pearls [25]. However, it is the unusual appearance of the central feature in this pearl prompted us to conduct further experiments to characterize the spherulitic material, which is the focus of this study. The granular morphology of the pearl’s center structure highly resemble the high-magnesium calcite synthesized via polymer-stabilized amorphous calcium magnesium carbonate (ACMC) precursors under mild conditions [26] or disordered dolomite created via aerobic bacteria biomasses [27] reported previously.

### Raman spectroscopic analyses

**Fig 3:**
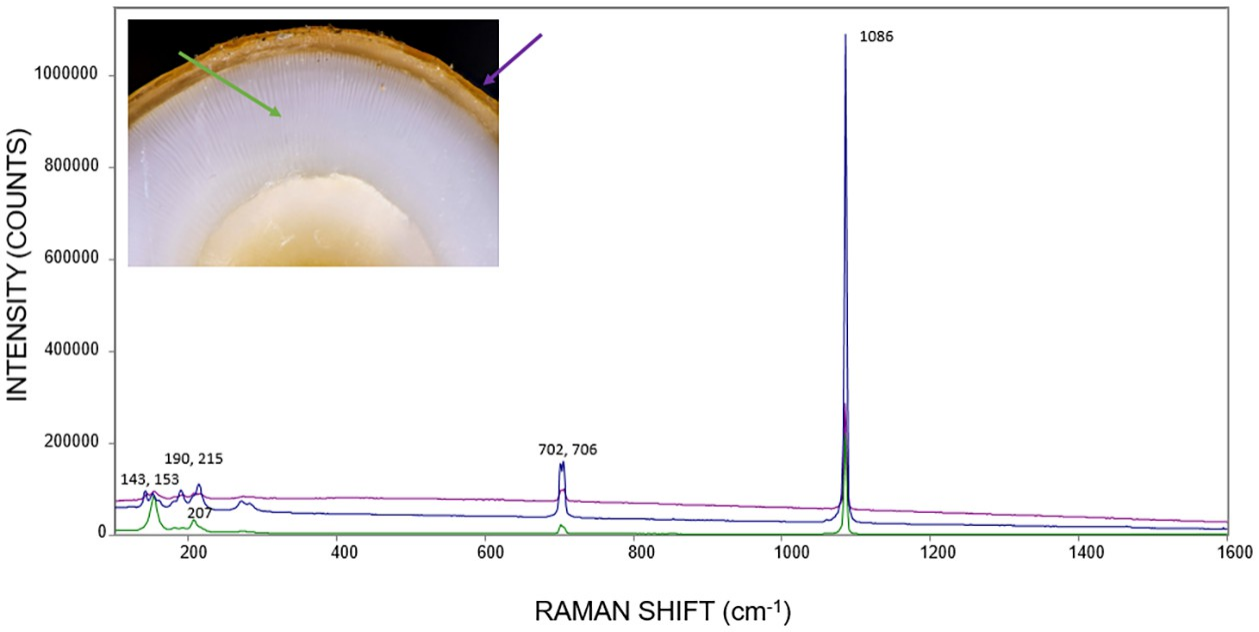
Raman spectra collected on the brown surface (spectrum in purple and also indicated with purple arrow in the image), the white area of the cross-section (spectrum in green and indicated with green arrow in the image) were concluded to be aragonite, similar to the typical aragonite found in nacreous pearl (spectrum in blue).

The Raman spectroscopy analyses were performed on three main areas of the sample: brown surface, white cross section, and brown granular center. Both surface and white region of the cross section showed typical Raman shifts for aragonite, with series of Raman peaks below 300 cm^-1^ that correspond to translational (T) and librational (L) lattice mode from the external vibration of CO_3_^2−^; a doublet at 702 and 706 cm^− 1^ which are assigned to in-plane bending (ʋ4) from internal vibration mode of CO_3_^2-^; and a major peak at 1086 cm^-1^ that correspond to symmetric stretching (ʋ1) from internal vibration mode of CO_3_^2−^. Previous studies suggest that shifts in Raman peak heights at lower wavenumber peaks in carbonates are signs of shifting crystallographic orientation within the shell or between crystallites [28]. This could explain the different patterns shown at the lower wavenumber region for these aragonite spectra. Both aragonite spectra from the brown surface and the white cross section, as well as an aragonite spectrum collected from a typical nacreous pearl for comparison were shown in Fig 3, and the white cross section coincides with flame structure presented in the area (inserted image in Fig 3), as various flame patterns displayed on surface of some non-nacreous pearls are the result of the cross-lamellar microstructure of aragonite laths, or fibers.

**Fig 4:**
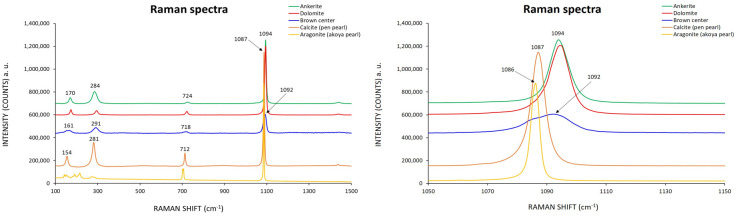
Raman spectrum collected on the brown center region of the sample showed series of peaks that do not fully conform to the typical aragonite or calcite structures found in pearls, but roughly matched with two carbonate minerals: Ankerite and dolomite, with some peak positions being shifted.

Raman spectra collected on brown granular center region showed peaks centered at 161 (T), 291 (L), 718 (ʋ4) and 1092 (ʋ1) cm^-1^ that do not fully conform to spectral characteristics of any CaCO_3_ polymorphs typically found in pearls, such as aragonite and calcite. Interpretation of these Raman patterns with RRUFF reference database matched with two carbonate minerals: ankerite (RRUFF ID: R050181.3) and dolomite (RRUFF ID: R050370.3) [29], however some peak positions are not entirely identical. Ankerite [Ca(Fe,Mn)(CO_3_)_2_] spectrum is characterized by the peaks at 170 (T), 284 (L), 724 (ʋ4), and 1094 (ʋ1) cm^-1^, and the Raman peaks of dolomite [CaMg(CO_3_)_2_] are located at 171 (T), 291 (L), 723 (ʋ4), and 1094 (ʋ1) cm^-1^. Fig 4 provides an overview of the different Raman peak shifts found in aragonite, calcite, ankerite, dolomite, and the center region of the *Cassis* pearl.

The main Raman band detected in the center brown region of our sample was also similar to the dolomite sample reported in a previous study [30] in 1200–600 cm^-1^ region but slightly different in 500–100 cm^-1^ region. Blue shift on ʋ1 Raman peak of dolomite comparing to calcite or aragonite is expected as the shorter Mg–O bonds cause a greater change in carbonate C–O vibrational frequency. Shifts of the band positions were also found to be related to variations in chemical compositions and crystal structures of different carbonate types and polymorphs. A study by Borromeo et al. [31] showed that the positions of vibrational modes are directly correlated to the amount of magnesium (Mg) in the crystal lattice of calcite, as well as other carbonate minerals that containing Mg. The Mg^2+^ ion substitutes for the Ca^2+^ ion in random and disordered ways in the lattice, and increasing concentrations of Mg is in accordance with shifted peak positions to higher wavenumbers. This suggests that the brown granular center of the *Cassis* pearl is not a pure CaCO_3_ biomineral, and it potentially contains higher concentrations of Mg in its structure.

**Fig 5:**
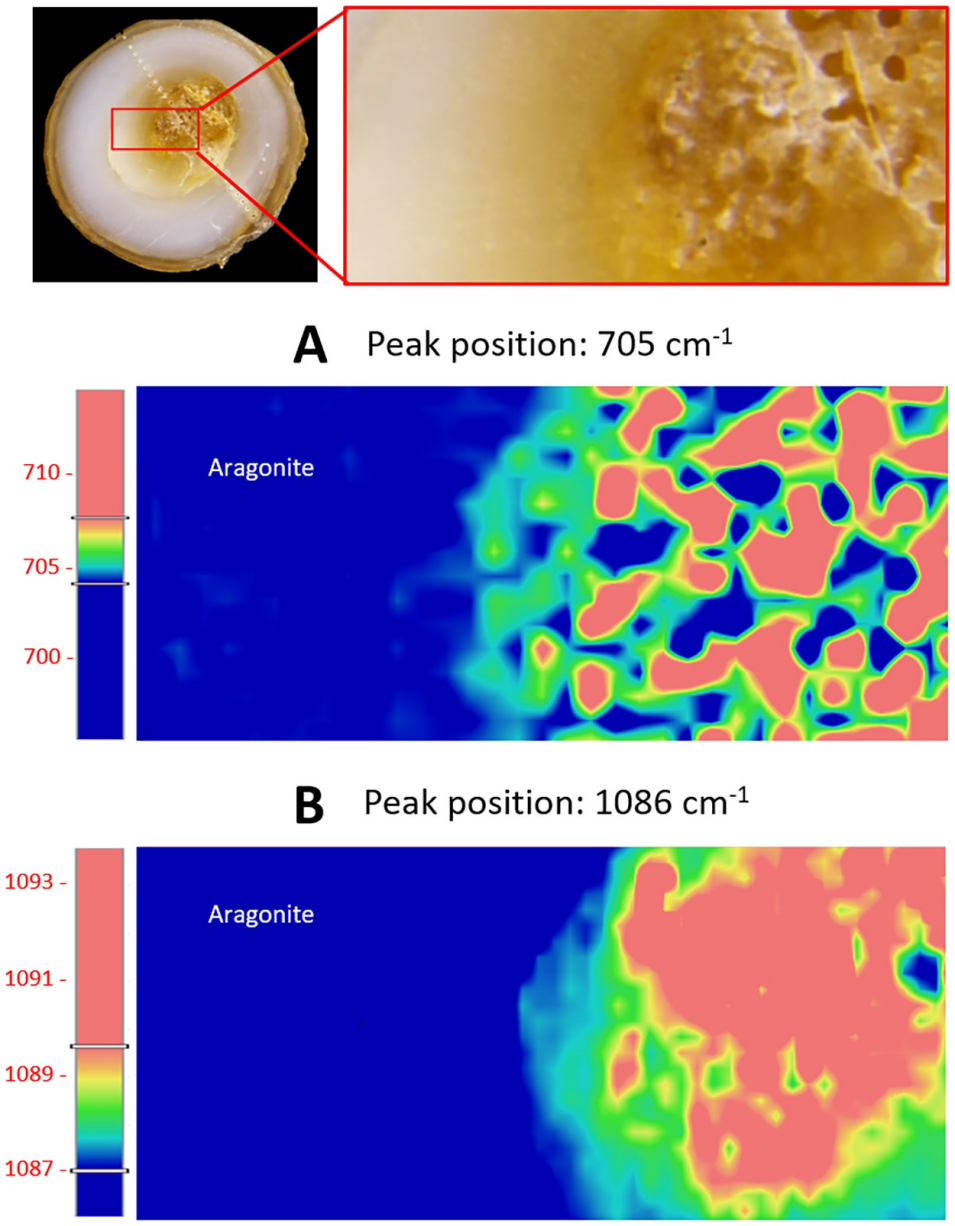
Raman maps collected using the 785 nm laser at room temperature showed intensity variations of the aragonite band at 705 cm^−^1 (A) and 1086 cm^−^1 (B). Both maps indicated that the central region of the Cassis pearl contained potentially high-Mg carbonate with characteristic peaks shifted to higher wavenumbers.

Raman and photoluminescence mapping have been proved to be particularly useful in many gem identification applications, including pearls [32]. The Raman maps shown in Fig 5A displayed intensity variation at 705 cm^−1^, which is a characteristic peak for aragonite. A low intensity zone (dark blue) indicated aragonite area. The higher intensity zones (green and yellow to pink) indicated a mixture of aragonite and high-Mg carbonates which were assigned based on the presence of the aragonite peak at 706 cm^-1^ and the high-Mg carbonate peak at 717 cm^-1^. The region between aragonite and high-Mg carbonates cannot be well-separated using the 705 cm^−1^ peak positon, instead, peak position at 1086 cm^-1^ was used (Fig 5B). The mapping image suggested that the brown center is dominated by high-Mg carbonates, with their symmetric stretching (ʋ1) peaks being shifted to higher wavenumbers, while the outer regions are mostly made of aragonite. It is likely that the center region is composed of carbonate polymorph that contains higher amount of Mg, yet it needs to be confirmed by more definitive method such as XRD analysis, which will be described in the next section.

### X-ray diffraction analyses

**Fig 6:**
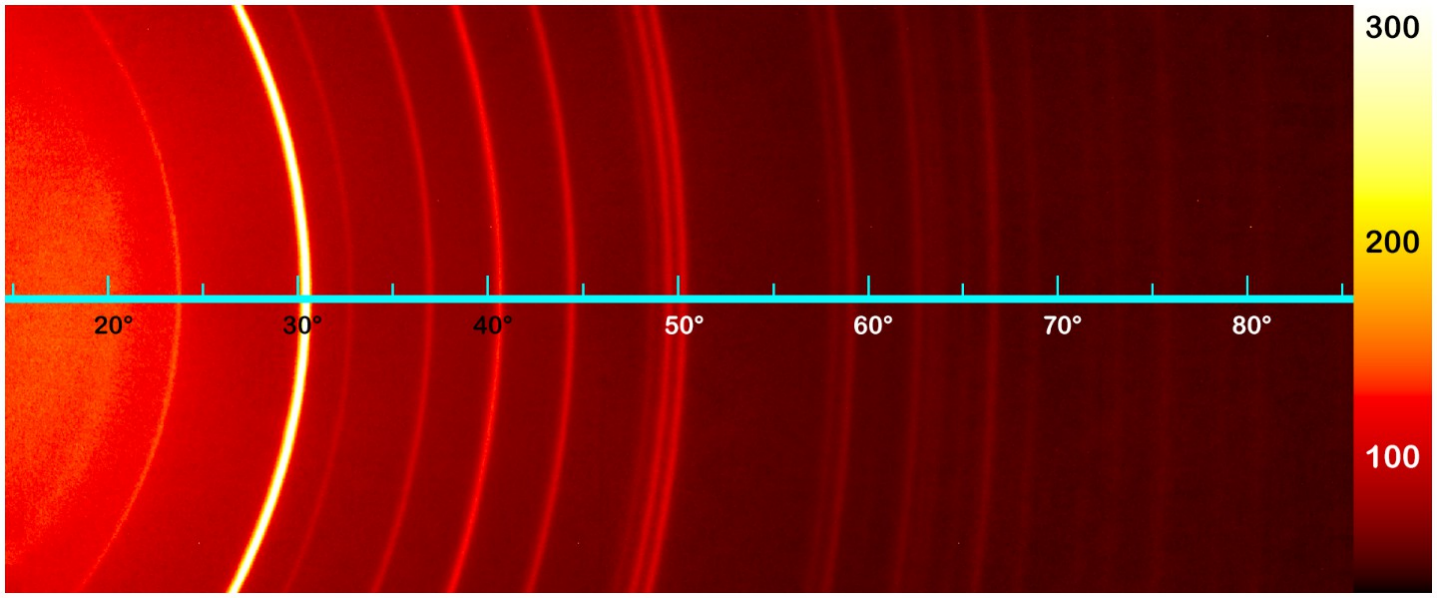
The diffraction pattern of the fragment separated from the center of the pearl. The image is merged from three unwarped frames taken at 2θ = 30°, 50°, 70°.

The small fragment taken from the center of the pearl was analyzed by XRD technique. The unwarped image merged from the three frames is shown in Fig 6. Some experiment and refinement details can be found in Table 1. The data collected on the fragment of the pearl displayed sharp and homogeneous Debye rings in the diffraction pattern, which indicated the φ+ω rotation has randomized the sample enough to produce a powder diffraction pattern with reliable intensities for Rietveld refinement. The refinement was initiated with a calcite structure. The lattice parameters were refined first using the Le Bail algorithm, before the atom positions were relaxed in the Rietveld refinement. Mg was then added to the cation site of the structure, with the total occupancy of the site constrained to 1. The atomic displacement parameters (ADPs) were relaxed to anisotropic for Ca, Mg and O atoms. The ADPs of the C atom were kept isotropic to make sure all the parameters in the refinement are realistic.

**Table 1:** Experimental and refinement details for the XRD data from the pearl fragment.

Chemical formula		Ca_0.53_Mg_0.47_CO_3_
Crystal system		Trigonal
Space group		$R\bar{3}c$
Unit cell parameter	*a* (Å)	4.85473(18)
	*b* (Å)	4.85473(18)
	*c* (Å)	16.3561(9)
	α (°)	90
	β (°)	90
	γ (°)	120
	*V* (Å^3^)	333.84(3)
*Z*		6
*D*_*x*_ (Mg m^-3^)		2.766
Scan		ω+φ rotation
Exposure Time		600s/frame
2θ values (°)	max	85
	min	15
	step	0.01
*R*_p_, *wR*_p_, *wR*(*F*^2^)		0.022, 0.029, 0.07
No. of parameters		15
No. of constraints		2
(Δ/σ)_max_		0.012

**Fig 7:**
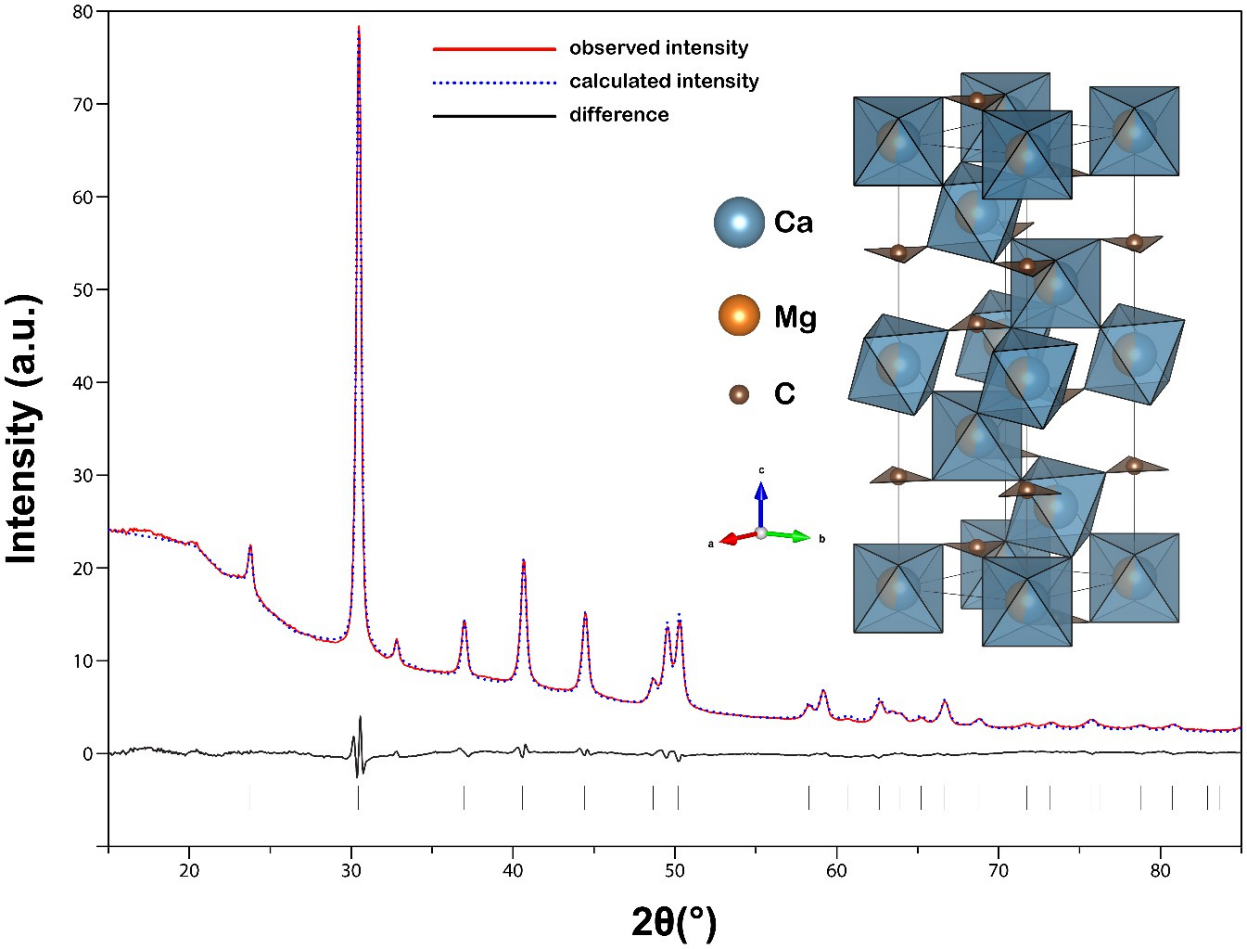
The structure refinement result from the XRD data collected on the pearl fragment. The final structure model is also presented. The calculated diffraction pattern from the structure model is plotted along with the collected data.

The final refinement result is shown in Fig 7. The resulting structure, which is also plotted in Fig 7, is a completely disordered dolomite with a space group symmetry of $\text{R}\bar{3}\text{c}$ and a chemical formula of Ca_0.53_Mg_0.47_CO_3_. The atomic coordinates and displacement parameters of the refined structure are listed in Table 2. No “b” reflections (l = 2n+1) associated with cation ordering was observed in the diffraction pattern. The absence of “b” reflections alone, however, is not enough to prove complete disordering in the structure, as Fang and Xu [33] showed that some weakly ordered protodolomite can display no visible “b” reflections in the XRD pattern. The combination of chemical composition and lattice parameters provides a better way to quantify the ordering state of the dolomite structure, as proposed by Fang and Xu. The d_104_ value of the refined structure (2.93Å) is the maximum possible value for the composition of Ca_0.53_Mg_0.47_CO_3_, corresponding to a completely disordered structure. The unit cell length a, c and the composition from the refinement also follows the relationship for disordered structure published by Zhang et al. [34].

**Table 2:** The atomic coordinates and displacement parameters of the refined structure from the XRD data.

	Occ.	x	y	z	U_eq_	U_11_	U_22_	U_33_	U_12_	U_13_	U_23_
Ca	0.530(8)	0	0	0	0.0238 (4)	0.0243 (5)	0.0243 (5)	0.0229 (7)	0.0121 (2)	0	0
Mg	0.470(8)										
C	1	0	0	¼	0.0218 (13)	N/A					
O	1	0.2661 (2)	0	¼	0.0478 (10)	0.0172 (10)	0.0639 (13)	0.0778 (17)	0.0319 (7)	-0.0025 (4)	-0.0051 (9)

**Fig 8:**
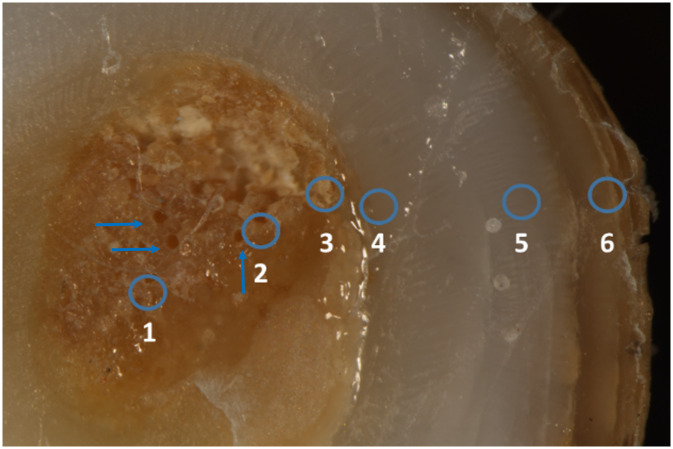
Microscopic image of the cross section and the spots for in-situ XRD analyses (circled areas). In the image a few ICPMS spots (indicated by arrows) can also be seen.

**Fig 9:**
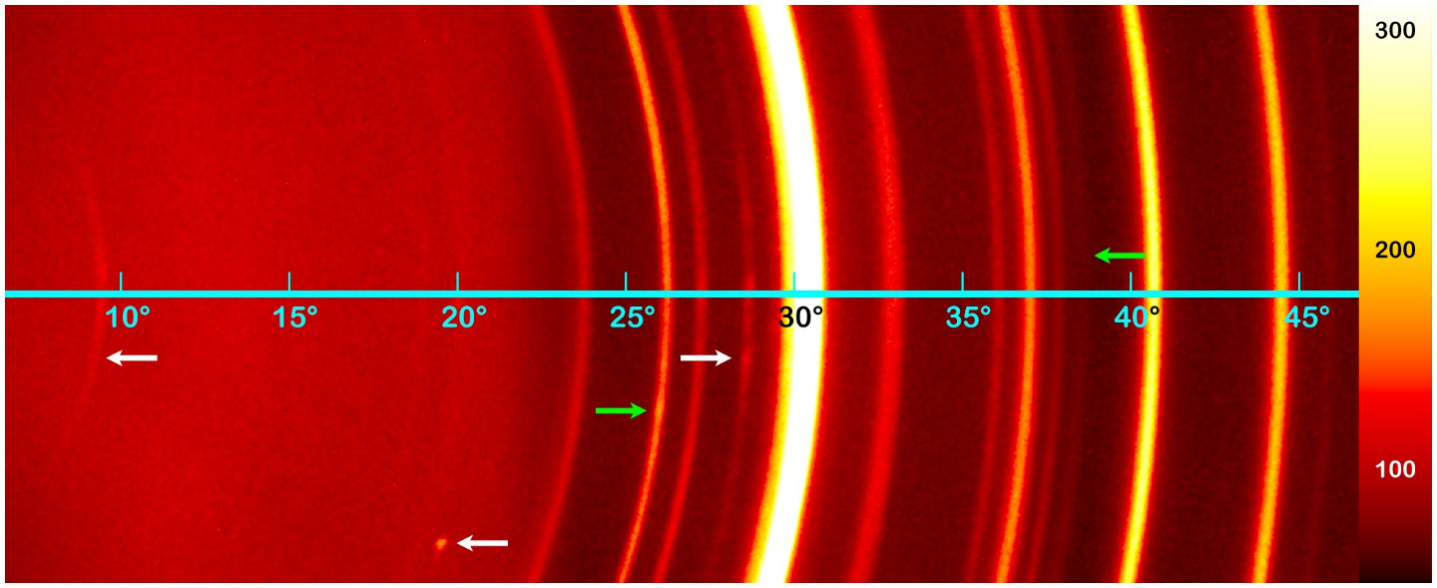
Part of the diffraction pattern collected on spot 1 marked in Fig 8. Some Debye rings showed obvious heterogeneity (marked by arrows), indicating possible preferred orientations of the mineral crystals in the pearl. Some weak reflections that did not match any carbonate phases can be observed in the core of the pearl sample (white arrows), which probably were from some organic material as suggested by the large unit cell.

The spots where in-situ XRD data were collected are marked in Fig 8, which have been numbered from the center to the edge. Part of the unwarped merged image from spot 1 is shown in Fig 9. Limited by the geometry of in-situ data collection, the pearl sample can only rotate perpendicular to the surface, around the spot being analyzed. As a result, obvious heterogeneity can be observed in the Debye rings (marked by arrows in Fig 9), which means the orientations in the sample were not randomized enough to produce an accurate powder diffraction pattern. This was partly due to the small numbers of crystals within the area analyzed, but may also reflect the preferably orientated crystals during the pearl growth. Because the X-ray hits the pearl surface at an angle, the interaction area would be significantly elongated along the beam direction. The X-ray also has strong penetration power, and would be diffracted by material beneath the surface. These are the complications introduced by the in-situ data collection experiment, which would alter the exact shape and position of the Debye rings in the diffraction pattern. This means an accurate analysis of the lattice parameters of the crystals is not possible without a calibration of the exact diffraction geometry, let alone a quantitative analysis of the mineral composition of each spot. Therefore, only qualitative phase composition can be acquired from the in-situ diffraction data.

**Fig 10:**
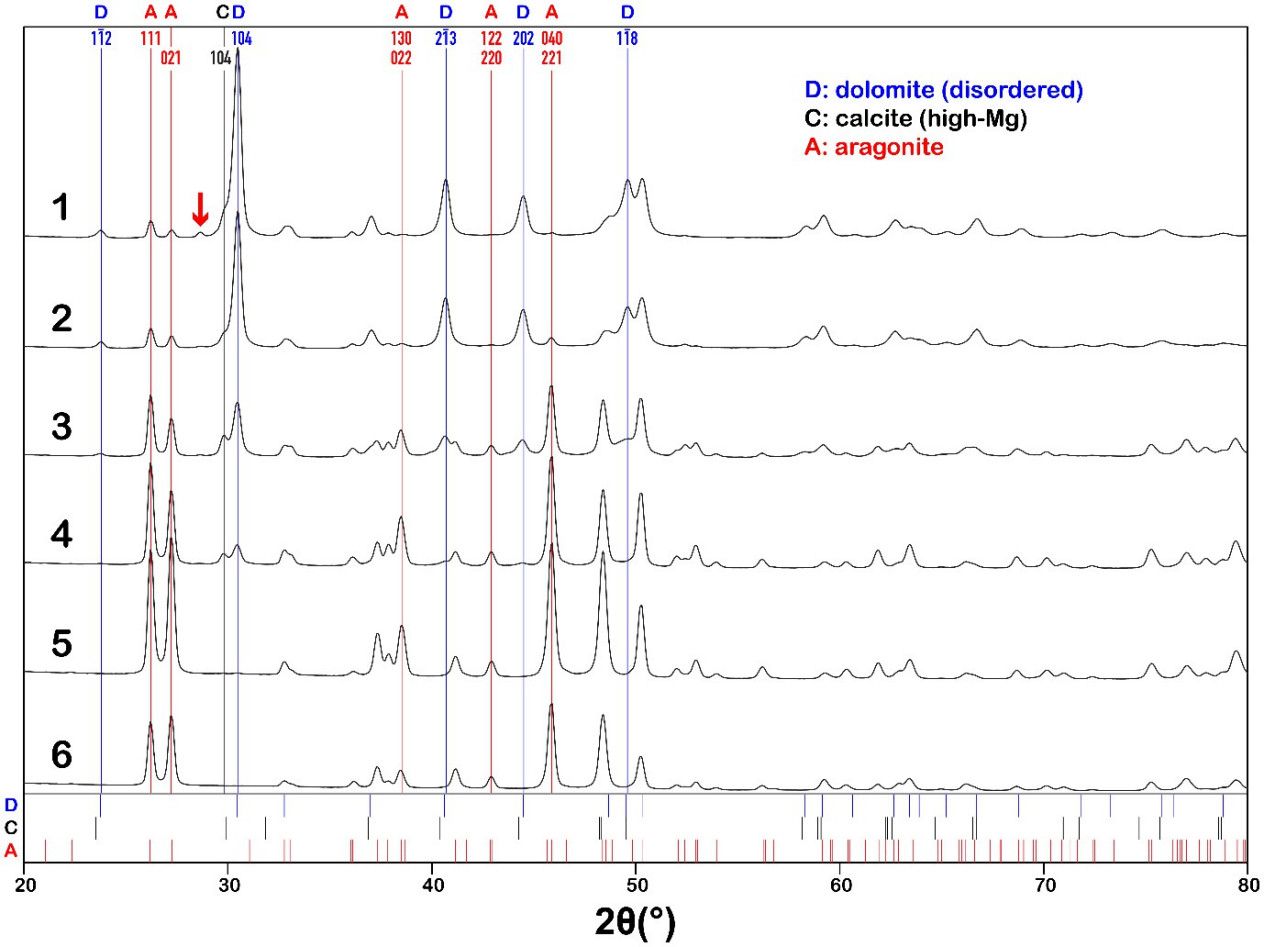
The XRD patterns integrated from the Debye rings collected in-situ on the pearl sample. The patterns were labelled by the spot numbers as shown in Fig 8. The reference diffraction peak positions for three phases (disordered dolomite, high-Mg calcite and aragonite) were marked at the bottom. Some representative peaks of each phase were also marked with vertical lines to show the change in phase compositions in the different zones of the pearl sample.

The integrated powder diffraction patterns from the in-situ data are shown in Fig 10. Three different phases were identified in the sample: disordered dolomite, high-magnesium calcite and aragonite. The “high-magnesium” composition of the calcite phase (>10 mol% MgCO_3_) was deduced from the relatively small (2.99Å) d_104_ value. A few very weak peaks that did not match any of the carbonate phases were detected in the core of the pearl sample (white arrows in Fig 9 and red arrow in Fig 10), which were probably from organic material, suggested by the large unit cell (first peak appeared below 10°). The results confirmed that the core of the pearl (spot 1 and 2) was dominated by disordered dolomite, yet small amount of aragonite and high-Mg calcite have also been detected. The white region and brown crust of the cross section were composed of only aragonite, with no detectable signal from any other phases. The decrease in absolute intensity from spot 5 to spot 6 was also an artifact caused by spot 6 being too close to the edge.

### Chemical analyses

**Fig 11:**
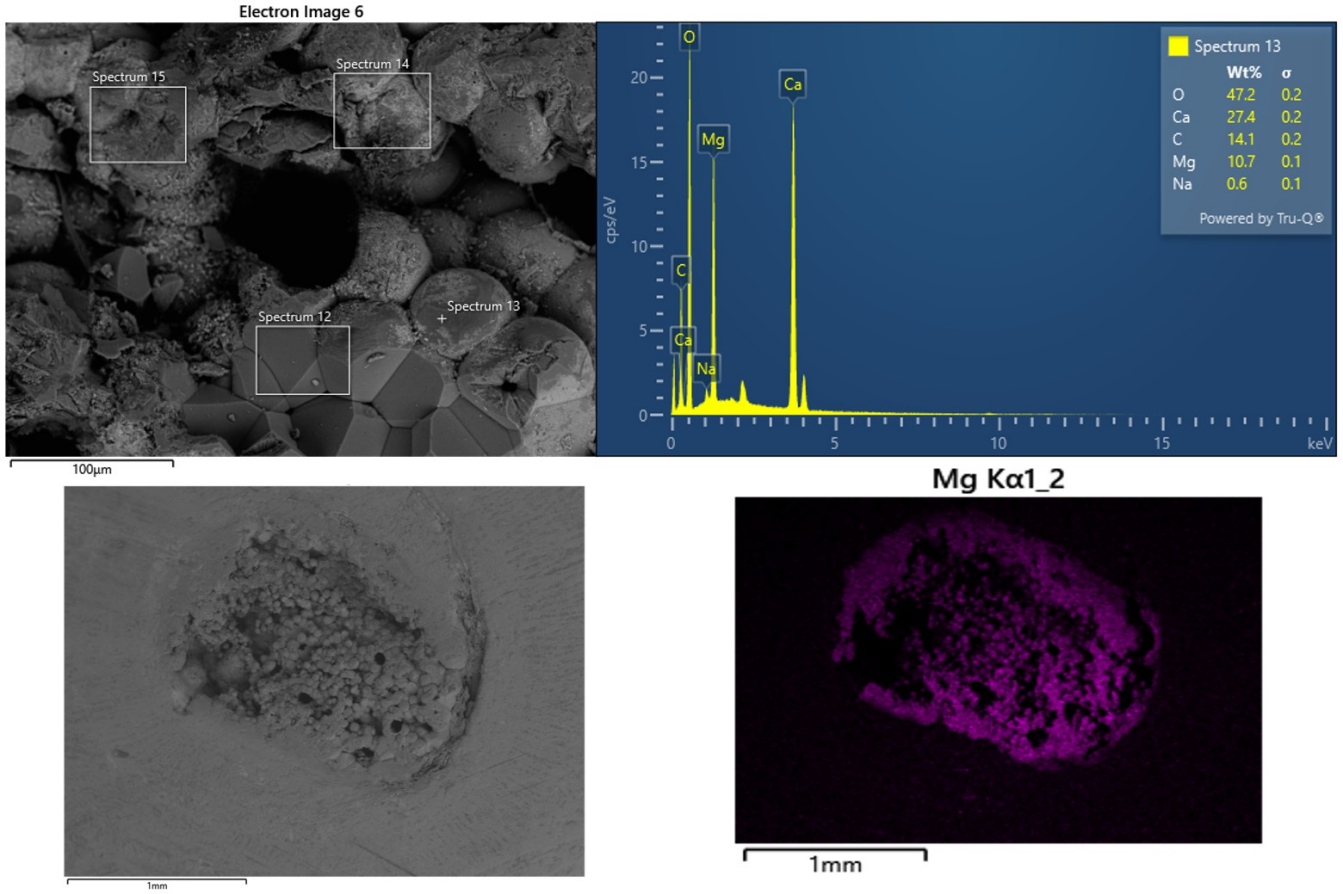
SEM image showing the areas and spot of EDS chemical analyses in the center of the pearl (top left), one representative EDS data spot (top right), and EDS mapping of the entire center region (bottom left and right) on Mg concentration showing elevated Mg content in the center of the pearl.

The concentrations of major and minor elements detected using ICPMS on six spots in the center region of the pearl are listed in Table 3. The main element of interest for this study is Mg, which could be used to support the result of XRD experiment: the presence of disordered dolomite in the center of this *Cassis* pearl. All six spots showed elevated Mg concentrations, with MgO concentration ranged from 9.30 to 13.03 weight percent, and Mg atomic proportions ranged from 0.2233 to 0.3086. In contrary, the white outer region of the pearl contained significantly less, trace amount of Mg (from 200 to 2000 ppmw). In addition, EDS mapping experiments confirmed that the high concentrations of Mg was only found in the central region of the pearl, as shown in Fig 11. Varying amount of Mg concentrations were detected in the center region, up to 10.7 weight percent. While the theoretical Mg concentration on the disordered dolomite calculated based on the XRD result is 12.32 weight percent, our experimental result was very close to the calculated result. These data agreed with the conclusion that the central region is composed of a mixture of dolomite, high Mg-calcite and aragonite. Lower concentrations of Mg were detected likely ascribed to heterogeneity of the pearl’s central region and the averaging effect of the spots being analyzed. The transition from disordered dolomite core to the aragonite-only mantle was rather sharp as shown by the EDS map, which means the seemingly gradual transition of the in-situ XRD data in Fig 10 is caused by an averaging effect of X-ray interacting with a larger area than marked in Fig 8.

**Table 3:** Concentrations of major and minor elements detected using ICPMS on six spots in the center region of the pearl.

Cassis pearl sample						
	spot 1	spot 2	spot 3	spot 4	spot 5	spot 6
Wt. % Oxides						
Na_2_O	1.13	0.95	1.05	1.19	1.37	1.11
MgO	9.66	9.46	9.30	10.48	13.03	10.27
CaO	43.13	43.66	43.75	42.17	38.96	42.54
SrO	0.40	0.34	0.35	0.34	0.37	0.34
CO_2_*	45.53	45.50	45.46	45.68	46.11	45.63
Others	0.14	0.09	0.09	0.14	0.16	0.11
Total	100	100	100	100	100	100
Atomic proportions on the basis of one carbon atom and three oxygen atoms						
Na	0.0353	0.0297	0.0329	0.0369	0.0421	0.0344
Mg	0.2317	0.2270	0.2233	0.2506	0.3086	0.2458
Ca	0.7434	0.7530	0.7551	0.7245	0.6631	0.7315
Sr	0.0038	0.0032	0.0032	0.0032	0.0034	0.0032
Total	1.0141	1.0129	1.0145	1.0153	1.0172	1.0149

*Carbon dioxide value is from calculation where 1 carbon atom per formula unit is assumed.

## Conclusion

Recent studies have shown that disordered dolomite can be precipitated at room temperature with the presence of catalysts including dioxane, polysaccharides, exopolymeric substances (EPSs), ethanol, and aqueous Si(OH)_4_ [35–39]. It is possible that the organic material created by the mollusk could act as catalysts during the formation of the disordered dolomite in the center of the pearl. The brown granular core of the pearl is obviously off-center relative to the concentric layering of the brown crust and the white mantle, suggesting it may be partially replacing the white aragonite layers. This means the disordered dolomite may be formed during (or even after) the formation of the pearl. It is hard to tell what the original irritant causing the formation of this pearl, but a Mg-rich liquid was likely present in its center. The precipitation of disordered dolomite could be triggered by the oversaturation of carbonate in this enclosed region. The spheroidal shape (commonly called spherulite) is very common for carbonate precipitates [38–40], which indicates fast precipitation from highly non-equilibrium conditions [41]. The eccentricity of the brown granular core is probably controlled by gravity (relative to the original orientation of the pearl during its formation). It is quite possible that before the pearl was cut open, the porous core still contained some liquid, which was immediately lost during the wet cutting process.

While many physical and biological factors play important roles in biomineralization of molluscan shells and the reasons for their varying microstructures are not completely clear to us [42], biomineralization of pearls has been even less understood, especially for natural pearls. Dolomite has been reportedly found in a Japanese akoya cultured pearl previously [43], which the authors used the dissolution rate of Mg to deduce the presence of dolomite in the pearl and the oyster shell studied. However, no signal of dolomite could be observed in the XRD data presented in that study, and the texture of carbonate as well as ordering state could all potentially affect the dissolution process, which was not taken into consideration by the method applied. Prior findings of dolomite in molluscan shells are also very limited in the literature [44, 45]. To our best knowledge, this is the first time disordered dolomite has been directly detected inside of a natural pearl. It provided a rare opportunity and invaluable insight into what natural biomineralization process and pearl formation could happen. It is likely that more interesting compositions at the core of pearls are waiting to be discovered, which are still widely unknown and waiting to be explored.
